# Molecular Detection Assays for Rapid Field-Detection of Rice Sheath Blight

**DOI:** 10.3389/fpls.2020.552916

**Published:** 2021-01-11

**Authors:** Ying-Hong Lin, Shih-Mao Shen, Chen-Jie Wen, Yi-Jia Lin, Tsai-De Chang, Sheng-Chi Chu

**Affiliations:** ^1^Department of Plant Medicine, National Pingtung University of Science and Technology, Pingtung, Taiwan; ^2^Plant Medicine Teaching Hospital, General Research Service Center, National Pingtung University of Science and Technology, Pingtung, Taiwan; ^3^Biological Control Branch Station, Miaoli District Agricultural Research and Extension Station, Council of Agriculture, Miaoli, Taiwan

**Keywords:** *Rhizoctonia solani*, polymerase chain reaction, SYBR green-based real-time PCR, TaqMan probe-based real-time PCR, fast detection

## Abstract

*Rhizoctonia solani* (Rs), a soil-borne fungal pathogen, can result in rice sheath blight (ShB), which causes yield loss. To prevent outbreaks of ShB and enhance the sustainability of rice production, it is critical to develop a rapid ShB detection method for specific, fast, and on-site disease management. In this study, a reagent for the rapid extraction of this pathogen was developed for on-site detection. The specificity and sensitivity of a novel SMS RS1-F/SMS RS1-R primer set and a ITS1/GMRS-3 reference primer set were tested, while four different extraction protocols for ShB were developed. Moreover, intraday and interday assays were performed to evaluate the reproducibility of the detection methods developed. The results indicated that all of the developed protocols are suitable for use in detecting ShB. In addition, all the samples of infected rice yielded positive Rs detection results when subjected to TaqMan probe-based real-time PCR and SYBR green-based real-time PCR (SMS RS1-F/SMS RS1-R) tests in which automatic magnetic bead-based DNA extraction was performed. These results indicated that the two molecular detection protocols were suitable for the field diagnosis of ShB for all asymptomatic and symptomatic rice samples.

## Introduction

Paddy rice (*Oryza sativa*), which is one of the world’s main food crops, takes up nearly 11% of the arable land worldwide, in addition to being the food that the largest proportion of people eat as their primary food (FAOSTAT, 2019). According to data from the FAO (Food and Agriculture Organization of the United Nations), the global yield of rice is nearly 782 million metric tons per year. During the cropping period, the plant may be attacked by various pathogens, including *Pyricularia oryzae, Bipolaris oryzae, Rhizoctonia solani, Fusarium fujikuroi*, and *Xanthomonas oryzae* pv. *oryzae* (Lu et al., 2014).

*Rhizoctonia solani* is a devastating soil-borne fungus which is classified into fourteen reproductively incompatible anastomosis groups (AGs). One of these, AG1, can be further subdivided into four intraspecific groups (ISGs), AG1-IA∼AG1-ID. *R. solani* AG1-IA and AG1-IB can infect some asterids and soybean (Hane et al., 2014), lead to rice sheath blight (ShB), and has been the most important factor in limiting rice production over the past two decades (Chen et al., 2012; Susheela and Reddy, 2013; Arakawa and Inagaki, 2014). *R. solani* can be propagated by sclerotia contained in irrigation water and soil, and survives for roughly 2 years in the land (Handiseni et al., 2015). The counterpart was already known and reported in the 20th century, and the yield of paddy rice dropped a lot in the United States, Japan, and China (Ou, 1985; Groth, 2005; Prasad and Eizenga, 2008; Bernardes-de-Assis et al., 2009; Zhang et al., 2009; Chen et al., 2012). It has become the primary pathogen affecting rice production for now (Yuan et al., 2018). ShB, moreover, can result in a global food crisis if the sickness becomes more and more serious (Lu et al., 2014), and in any case, it is a limiting factor of rice yields. To develop a rapid method for detecting ShB, therefore, is vital and essential.

DNA probes and PCR (polymerase chain reaction) are widely used nowadays to detect plant diseases (Dubey et al., 2016). PCR is a technique with high efficiency, specificity, and sensitivity and can be used to detect the associated pathogens (Lin et al., 2009). Recently, it has become possible to design unique primers to pick up the ribosomal DNA (rDNA) of a pathogen by an internal transcribed spacer (ITS), and the use of this approach is of substantial help in differentiating among the different pathogens (White et al., 1990; Johanson et al., 1998). Moreover, the technique of rDNA analysis can also be used to analyze the genetics of fungal families (James et al., 2001). Some researchers have also used different types of PCR to analyze *R*. *solani* and other fungal species. For example, Johanson et al. (1998) designed various pairs of ITS primers (ITS1/GMRS-3, ITS1/GMRS-4, GMRO-3/R635, ITS1/GMROS-2, and GMROS-6/R635) to differentiate and detect *R*. *solani*, *R*. *oryzae*, and *R*. *oryzae*-*sativae*, while other researchers have used restriction fragment length polymorphism (RFLP) to mark the different AGs of *R*. *solani* (Bernardes-de-Assis et al., 2009; Çebi Kılıçoğlu and Özkoç, 2010). In addition, quantitative real-time polymerase chain reaction (qPCR) is a technique which has been widely used in detecting plant pathogens (Lievens et al., 2006; Sayler and Yang, 2007; Lin et al., 2013). The technique has been used for detection and quantification of *R*. *solani* AG-1 IA by Slayer and Yang from 2007. In that study, they used the tissue of rice infected by ShB to develop the technique, in addition to separating different groups of *R*. *solani* via ITS. Other researchers subsequently used this system to design a primer to detect the progression of ShB (Su′udi et al., 2013). Thus far, however, no researchers have used PCR or qPCR techniques to quantify sclerotia in the field. Nonetheless, if quantities of sclerotia could be measured, the chances of controlling the disease would be increased.

Extracting DNA is the critical step for molecular bioassays. In the past, DNA has usually been extracted by organic reagent extraction. The targeted DNA were extracted by the phenol/chloroform method and finally dissolved into a buffer (Murray and Thompson, 1980). Recently, column purification has served as a standard method for extracting DNA (Shi et al., 2018). In this approach, cells are destroyed by an anion surfactant and RNA are degraded with RNase, after which proteinase K is added to decompose proteins and increase the production rate of DNA. The resulting supernatant with DNA is added into a purification column, and the DNA is then collected on the filter membrane. Later, this DNA can be washed out by a buffer (Liu et al., 2000; Štorchová et al., 2000). Unfortunately, the DNA extraction methods mentioned above are not easy to use in the field; therefore, in this study, we developed a single buffer extraction method to cope with this shortcoming. This single buffer extraction method is an efficient, convenient, and time-saving way to extract DNA in the field.

We used the ITS gene sequence to design a specific primer for ShB to develop a new and fast way to detect *R. solani* in this study. To make sure of the specificity and sensitivity of the new method, we used PCR (Johanson et al., 1998) and qPCR, respectively. This study sought to establish a method for detecting ShB in the field. We focused on extracting nucleic acid and developing a fast extraction reagent to improve upon the techniques currently used for detecting ShB. We also performed intraday and interday assays (Skottrup et al., 2007) to ensure the stability of this new method. Randomly collected infected samples from field were analyzed by PCR, SYBR green-based qPCR, and TaqMan probe-based qPCR in a laboratory to ensure that the newly developed method is suitable for detecting ShB.

## Materials and Methods

### Growth of Fungal Species

Fungal rice pathogens confirmed by pathogenicity test on rice cultivar TNG67, included ten *R*. *solani* (Rs) isolates (rice ShB disease, PM-SMS-F001∼PM-SMS-F009, and PM-SMS-F013), five *Pyricularia oryzae* isolates (rice blast disease, PM-SMS-F016, PM-SMS-F017, PM-SMS-F018, PM-SMS-F021, and PM-SMS-F022), four *Bipolaris oryzae* isolates (rice brown spot disease, PM-SMS-F024, PM-SMS-F025, PM-SMS-F026, and PM-SMS-F027), and one *Fusarium verticillioides* isolate (rice bakanae disease, PM-YHL-F056), in this study (Table 1). The genomic DNA (gDNA) from other fungal pathogens, included *Colletotrichum gloeosporioides* (PM-TDC-F013), *C*. *lagenarium* (PM-LLH-F004), *F*. *oxysporum* f. sp. *cubense* (PM-YHL-F016 and PM-YJL-F040), *F*. *oxysporum* f. sp. *niveum* (PM-YHL-F045), *F*. *acuminatum* (PM-YHL-F018), *F*. *oxysporum* f. sp. *gladioli* (PM-YHL-F019), and *Alternaria alternata* (PM-TDC-F016), for comparison (Table 1). Sclerotium and hyphae of Rs and single spore cultures of the other fungal pathogens were grown on a PDA plate (200 g/L of potato extracts, 1% glucose, and 2% agar) in a growth chamber at 28 ± 2°C under 12 h/12 h cycles of light and darkness. After 7 days of incubation, the sclerotium or hyphae were collected for further DNA isolation.

**TABLE 1:** Isolates of plant pathogens used in this study and their PCR identification.

**Isolate code numbers**	**Diseases/species**	**Original hosts/tissues**	**Geographic locations**	**PCR-based identification**		
				**ITS1/ITS4^a^**	**SMS RS1-F/SMS RS1-R^b^**	**ITS1/GMRS-3^c^**
PM-SMS-F001	Rice sheath blight disease (ShB)/*Rhizoctonia solani* (Rs) AG1	Rice (*Oryza sativa* L.)/Sheath (S)	Pingtung, Taiwan	+	+	+
PM-SMS-F002	ShB/Rs AG1	Rice/S	Pingtung, Taiwan	+	+	+
PM-SMS-F003	ShB/Rs AG1	Rice/S	Pingtung, Taiwan	+	+	+
PM-SMS-F004	ShB/Rs AG1	Rice/S	Pingtung, Taiwan	+	+	+
PM-SMS-F005	ShB/Rs AG1	Rice/S	Tainan, Taiwan	+	+	+
PM-SMS-F006	ShB/Rs AG1	Rice/S	Tainan, Taiwan	+	+	+
PM-SMS-F007	ShB/Rs AG1	Rice/S	Tainan, Taiwan	+	+	+
PM-SMS-F008	ShB/Rs AG1	Rice/S	Tainan, Taiwan	+	+	+
PM-SMS-F009	ShB/Rs AG1	Rice/S	Tainan, Taiwan	+	+	+
PM-SMS-F013	ShB/Rs AG1	Rice/S	Yunlin, Taiwan	+	+	+
PM-SMS-F016	Rice blast disease (RB)*/Pyricularia oryzae* (Po)	Rice/Leaf (L)	Taitung, Taiwan	+	−	−
PM-SMS-F017	RB/Po	Rice/L	Yilan, Taiwan	+	−	−
PM-SMS-F018	RB/Po	Rice/L	Kaohsiung, Taiwan	+	−	−
PM-SMS-F021	RB/Po	Rice/L	Changhua, Taiwan	+	−	−
PM-SMS-F022	RB/Po	Rice/L	Miaoli, Taiwan	+	−	−
PM-SMS-F024	Rice brown spot disease (RBS)/*Bipolaris oryzae* (Bo)	Rice/L	Changhua, Taiwan	+	−	−
PM-SMS-F025	RBP/Bo	Rice/L	Changhua, Taiwan	+	−	−
PM-SMS-F026	RBP/Bo	Rice/L	Changhua, Taiwan	+	−	−
PM-SMS-F027	RBP/Bo	Rice/L	Nantou, Taiwan	+	−	−
PM-YHL-F056	Rice bakanae disease*/Fusarium verticillioides*	Rice/Stem	Taichung, Taiwan	+	−	−
PM-TDC-F013	*Colletotrichum gloeosporioides*	Melon (*Cucumis melo* L.)/L	Pingtung, Taiwan	+	−	−
PM-LLH-F004	*C. lagenarium*	Watermelon/L	Taichung, Taiwan	+	−	−
PM-YHL-F016	*F. oxysporum* f. sp. *cubense*	Banana (*Musa* sp.)/Pseudostem	Pingtung, Taiwan	+	−	−
PM-YJL-F040	*F. oxysporum* f. sp. *cubense*	Banana/Pseudostem	Pingtung, Taiwan	+	−	−
PM-YHL-F045	*F. oxysporum* f. sp. *niveum*	Watermelon (*Citrullus lanatus* (Thunb.) Matsum. and Nakai)	Taiwan	+	−	−
PM-YHL-F018	*F. acuminatum*	Bermuda grass (*Cynodon dactylon* (L.) Pers)	Taichung, Taiwan	+	−	−
PM-YHL-F019	*F. oxysporum* f. sp. *gladioli*	Gladiolus (*Gladiolus* sp.)	Taiwan	+	−	−
PM-TDC-F016	*Alternaria alternata*	Melon/Fruit	Kaohsiung, Taiwan	+	−	−

*^*a*^The conserved primer set ITS1/ITS4 was used to amplify and sequence the ∼500-bp rDNA region used for the identification of the internal transcribed spacers 1 (ITS1), 5.8S rDNA, and ITS2 of the fungal pathogens used in this study. ^*b*^The *Rhizoctonia solani*-specific primer set SMS RS1-F/SMS RS1-R designed in this study was used for the molecular detection assays of rice sheath blight. ^*c*^The *Rhizoctonia solani*-specific primer set GMRS-3/ITS1 designed by Johanson et al. (1998) was used for comparison with the primer set SMS RS1-F/SMS RS1-R.*

### Primer and TaqMan Probe Design

The Rs-specific primers for ShB detection were designed as SMS RS1-F/SMS RS1-R, which was modified from the research of Johanson et al. (1998). Also, an SMS RS-probe (5′-FAM-CCCTCCTGCCAAATT-BHQ-1-3′) was designed. The primers and probe were also checked by primer express 3.0, for GC content and Tm (melting temperature) value. Furthermore, Oligo 7 was used to ensure that it was not easy for duplex formation or hairpin formation to occur among the primers, probes, and DNA template. The conserved primer set ITS1/ITS4 was used to amplify and sequence the ∼500-bp rDNA regions, including the ITS1, 5.8S rDNA, and ITS2 (White et al., 1990), in order to identify the isolates tested in this study. Another Rs-specific primer set, ITS1/GMRS-3, which produced a 550-bp DNA fragment published previously by Johanson et al. (1998), was used to confirm the specificity of the Rs detection assay and for comparison with SMS RS1-F/SMS RS1-R in further molecular evaluation assays (including sensitivity, reproducibility, and field-*in*-*planta* detection assays). The sequences of the primer set ITS1/ITS4 (as PCR internal control), ITS1/GMRS-3, and SMS RS1-F/SMS RS1-R were listed in Table 2.

**TABLE 2:** Amplification primers used in this study.

**Associated pathogens**	**Amplification primers**		
	**Names**	**Sequences (5′-3′)**	**References**
*Rhizoctonia solani*	SMS RS1-F/SMS RS1-R	AACCTGCGGAAGGATCATTATTG/GGTGTGATGGATGAAAGAGAAGGT	This study
*R. solani*	ITS1/GMRS-3	AGTGGAACCAAGCATAACACT/TCCGTAGGTGAACCTGCGG	Johanson et al. (1998)
All fungal pathogens	ITS1/ITS4	TCCGTAGGTGAACCTGCGG/TCCTCCGCTTATTGATATGC	White et al. (1990)

### DNA Extraction Methods

Four DNA extraction methods (rapid DNA extraction, automatic magnetic bead-based DNA extraction, spin column-based DNA extraction, and CTAB/phenol/chloroform-based DNA extraction) were used in this study for comparisons. For rapid DNA extraction, the artificially Rs-inoculated and field-infected rice samples were washed, surface-sterilized with 1% sodium hypochlorite (NaHClO), rinsed in sterile water, and dried under a laminar flow hood to eliminate epiphytic microbes. The surface-sterilized rice leaf sheaths were cut into 1 cm^2^ sections (300 mg), put into a mortar and mill in 1.4 mL of lysis buffer (25 mM NaOH, 2 mM EDTA). After centrifugation at 6,000 × *g* for 1 min, the supernatant with gDNA was subjected to further molecular detection. Each rice sheath leaf (300 mg) was frozen in liquid nitrogen and finely ground using a mortar and mill. The other three DNA extraction protocols, which were based on the automatic silica-coated magnetic bead-based DNA extraction (taco mini Automatic Nucleic Acid Extraction System, GeneReach, United States), spin column-based DNA extraction (Viogene genomic mini kit, Viogene-BioTek, Taipei, Taiwan), and CTAB/phenol/chloroform-based DNA extraction methods (Porebski et al., 1997), were further carried out according to the manufacturers’ instructions and the protocol described by Porebski et al. (1997), respectively. Genomic DNA was dissolved in a 0.1 × TE buffer (1 mM Tris–HCl and 0.1 mM EDTA, pH 8.0) and stored at −20°C for further molecular detection assays.

### Primer Sensitivity Assays

Three PCR templates (mycelial, sclerotial gDNA and standard DNA) were used in primer sensitivity assays for comparisons. Fresh mycelia (100 mg) and sclerotia (100 mg) were frozen in liquid nitrogen and finely ground using a mortar and mill. The gDNA of the mycelia or sclerotia extracted by the spin column-based DNA extraction method were used for further primer sensitivity assays. To generate the standard templates, the 118 and 550-bp DNA sequences amplified by the Rs-specific primer sets SMS RS1-F/SMS RS1-R and ITS1/GMRS-3 were gel-purified, cloned into pGEM^®^ -T Easy vector (Promega Co., Madison, WI, United States), and sequenced, respectively. The copy number calculation of the standard templates was based on the concentrations determined by a SPECTROstar Nano spectrophotometer (BMG Labtech, Ortenberg, Germany). The standard templates were dissolved in a 0.1 × TE buffer and stored at −20°C for further primer sensitivity assays.

### Phylogenetic Analysis

The ten Rs isolates were used for ITS DNA sequencing with the primer sets ITS1/ITS4 and further phylogenetic analysis. ITS sequences of the isolates were aligned with available homologs from GenBank. Multiple sequence alignments were performed using the Clustal W multiple sequence alignment program of MEGA, version X (Tamura et al., 2007; Kumar et al., 2018). Neighbor-joining (NJ) tree was constructed using MEGA, version X (Tamura et al., 2007; Kumar et al., 2018). The NJ stability of the relationships were evaluated by a bootstrap analysis with 1,000 replications.

### Sampling Criteria of Infected Tissues

Rice plants were grown in a growth chamber (28°C, 12 h photoperiod) or under field conditions (35/24°C day/night temperature and 75/85% day/night relative humidity). To obtain uniform disease development, an artificial Rs-inoculation assay (10-week-old rice plants) was performed in the growth chamber (28°C, 12 h photoperiod) according to Park et al. (2008). The levels of scoring of ShB used were in accordance with those of the IRRI (1996) and Rodrigues et al. (2003a, b). The scoring scale ranged from 0 to 9 based on the lesions on the whole plant: 0 = no symptoms; 1 = injuries limited to the lower 20% of the plant height; 3 = lesions limited to the lower 20 to 30% of the plant height; 5 = lesions limited to the lower 31 to 45% of the plant height; 7 = injuries limited to the lower 46 to 65% of the plant height; 9 = lesions affecting more than 65% of the plant height. We collected 24 artificially Rs-inoculated rice samples in the growth chamber and 96 field-infected rice samples from 8 different fields that had been strongly affected by ShB. The rice samples showing varying symptoms were washed, surface-sterilized with 1% NaHClO, rinsed in sterile water, and dried under a laminar flow hood to eliminate epiphytic microbes. The surface-sterilized rice leaf sheaths were cut into 1 cm^2^ sections and put onto a PDA medium for a plate-out assay. Simultaneously, a piece of the rice leaf sheaths (0.3 g) surrounding each section was used for DNA extraction according to the DNA extraction methods. The DNA samples (50 ng) of symptomatic and symptomless leaf sheaths were used for further *in*-*planta* detection assays.

### Molecular Detection Assays

Three molecular techniques (conventional PCR, SYBR green-based real-time PCR and TaqMan probe-based real-time PCR; we refer hereafter to these as cPCR, SYBR-qPCR, and TaqMan-qPCR, respectively) were used with the primer sets SMS RS1-F/SMS RS1-R (designed in this study) and ITS1/GMRS-3 (Johanson et al., 1998) for *in*-*planta* detection assays for comparisons. For cPCR analysis, each 25 μl PCR mixture contained the tested templates, 1 × PCR Dye Master Mix II (GMbiolab Co., Ltd., Taichung, Taiwan), and 0.25 μM primers (SMS RS1-F/SMS RS1-R or ITS1/GMRS-3). The parameters for cPCR were denaturing at 95°C for 3 min, followed by 30 cycles of denaturing at 95°C for 30 s, annealing at 57°C for 30 s, and polymerizing at 72°C for 60 s, followed by a final extension at 72°C for 5 min. All PCRs were performed using a T100^TM^ Thermal Cycler (Bio-Rad Laboratories., Co., Ltd., Hercules, CA, United States). PCR products were subjected to electrophoresis in 2.0% agarose gels. The agarose gel was visualized, photographed, and analyzed by Gel DocTM EZ Imager (Bio-Rad Laboratories., Co., Ltd., Hercules, CA, United States). For SYBR-qPCR analysis, each 25 μl real-time PCR mixture contained the tested templates, 1× KAPA SYBR^®^ FAST qPCR Kit Master Mix Universal (Kapa Biosystems., Inc., Wilmington, MA, United States), and 0.25 μM primers (SMS RS1-F/SMS RS1-R or ITS1/GMRS-3). The parameters for SYBR-qPCR were 95°C for 5 min, followed by 40 cycles of 95°C for 10 s and 60°C for 20 s (annealing and polymerizing). After the real-time PCR, melting curves (65 to 99°C, raised 0.1°C every 1 s) of the PCR products were analyzed to verify their specificity. For TaqMan-qPCR analysis, each 25 μl real-time PCR mixture contained the tested templates, 1× KAPA Probe FAST qPCR Kit Master Mix Universal (Kapa Biosystems., Inc., Wilmington, MA, United States), 0.25 μM primers (SMS RS1-F/SMS RS1-R), and the SMS RS-probe (5′-FAM-CCCTCCTGCCAAATT-BHQ-1-3′). The parameters for TaqMan-qPCR were 95°C for 5 min, followed by 40 cycles of 95°C for 10 s and 60°C for 20 s (annealing and polymerizing). The two qPCR analyses were monitored on a CFX96 Touch^TM^ Real-Time PCR Detection System (Bio-Rad Laboratories., Co., Ltd., Hercules, CA, United States). The standard curve was created by plotting the target DNA amount against the threshold cycle (Ct) value exported from the CFX96 Touch^TM^. To ensure the reproducibility of the molecular detection assays, intraday (four assays on the same day) and interday (four assays on different days) validations were carried out according to the description by Skottrup et al. (2007) to evaluate experimental variation. We performed these intraday and interday assays, by four assays with three DNA extractions in each assay, on different diseased samples that were collected from the artificially Rs-inoculated rice.

## Results

### Testing the Specificity of the Primer Sets SMS RS1-F/SMS RS1-R and ITS1/GMRS-3

**FIGURE 1:**
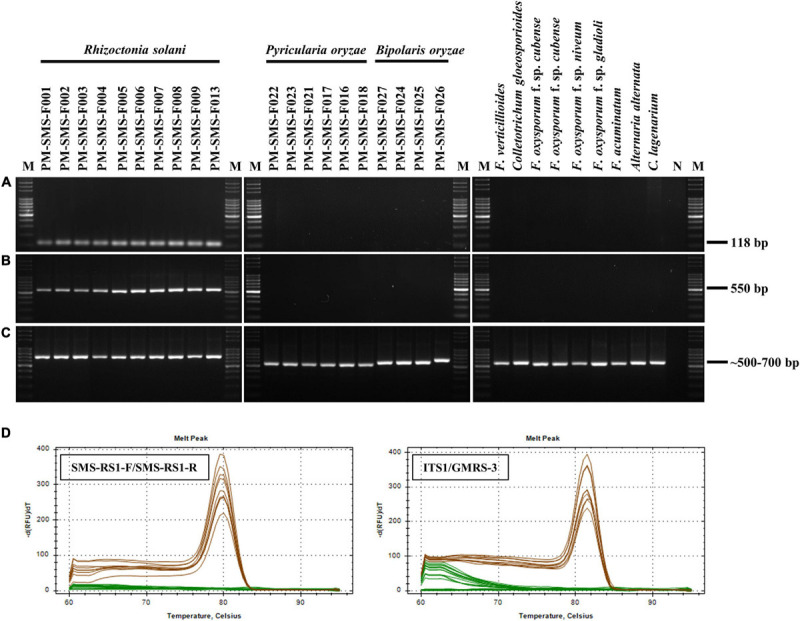
Specificity test results of the primer sets SMS RS1-F/SMS RS1-R **(A)** and ITS1/GMRS-3 **(B)**. A total of 10 ng samples of genomic DNA (gDNA) from each of 11 *Rhizoctonia solani* isolates (which cause rice sheath blight), 5 *Pyricularia oryzae* isolates (which cause rice blast disease), 4 *Bipolaris oryzae* isolates (which cause rice brown spot disease), 1 *Fusarium verticillioides* isolate (which causes rice bakanae disease), and 8 different plant pathogen isolates were used as PCR templates (all isolates as listed in Table 1). After PCR, melting curves (60 to 99°C) of the PCR products were analyzed to verify their specificity. PCR results of the primer set ITS1/ITS4 were used as PCR internal controls **(C)**. A single and prominent peak (at 79.5 or 81.5°C) of Rs amplicons (brown lines) was presence in dissociation curves **(D)**. N: negative control using sterile ddH_2_O as the PCR template; M: molecular markers of Gen-100 DNA ladder.

**FIGURE 2:**
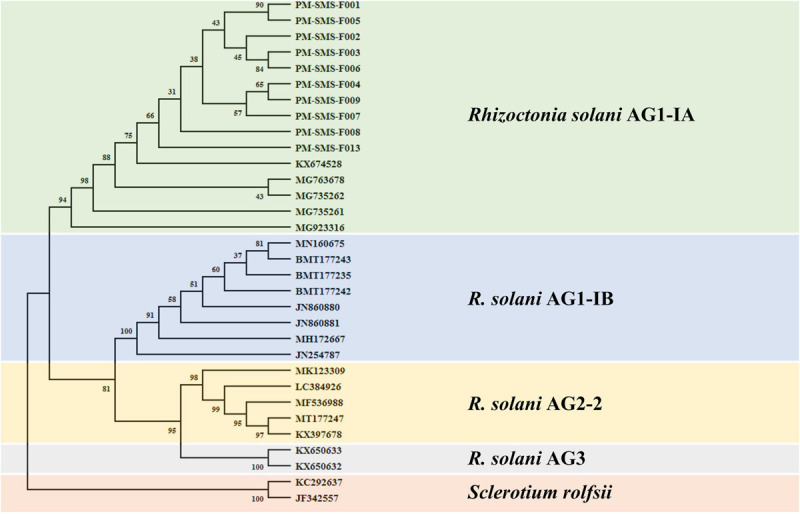
Phylogenetic tree generated by neighbor-joining method based on the rRNA gene locus sequences coding for ITS showing the phylogenetic relationships of *Rhizoctonia solani* (AG1-IA, AG1-IB, AG2-2, and AG3). Two isolates of *Sclerotium rolfsii* was used as the outgroup. The bootstrap consensus tree inferred from 1000 replicates is taken to represent the evolutionary history of the taxa analyzed. Bootstrap values of the internal branches are indicated. Evolutionary analyses were conducted in MEGA X.

Only *R*. *solani* can be amplified and visualized by the bands at 118 and 550 bp when using the primer pairs ITS1/GMRS-3 and SMS RS1-F/SMS RS1-R (Figure 1), whereas, other pathogens, such as *P. oryzae*, *B. oryzae*, *F*. *verticillioides*, *C*. *gloeosporioides*, *C*. *lagenarium*, *F*. *oxysporum* f. sp. *cubense*, *F*. *oxysporum* f. sp. *niveum*, *F*. *acuminatum*, *F*. *oxysporum* f. sp. *gladioli*, and *A. alternata*, do not elicit the same results as *R*. *solani*. Moreover, these *R*. *solani* isolates were identified as *R*. *solani* AG1-IA based on the phylogenetic analysis of their ITS sequence (Figure 2). Taken together, these results prove that the primer we designed, SMS RS-1F/SMS RS1-R, and the reference primer ITS1/GMRS-3 (Johanson et al., 1998) have specificity for the pathogen of ShB.

### Testing the Sensitivity of the Primer Sets SMS RS1-F/SMS RS1-R and ITS1/GMRS-3

**FIGURE 3:**
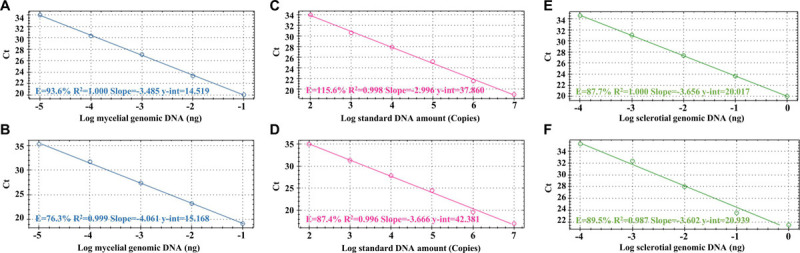
Detection sensitivity results of the SYBR green-based real-time PCR (SYBR-qPCR) assays with the primer sets SMS RS1-F/SMS RS1-R **(A,C,E)** and ITS1/GMRS-3 **(B,D,F)**. Serial dilutions of *Rhizoctonia solani* SMS-F013 mycelial DNA ranging from 10^–1^ to 10^–5^ ng **(A,B)**, standard DNA ranging from 10^7^ to 10^2^ **(C,D)**, and sclerotial DNA ranging from 10 to 10^–4^ ng **(E,F)** were used as SYBR-qPCR templates. The standard curves were created by plotting the template amount against the threshold cycle (Ct) value.

After the specificity of the primers was proved, we used these primers to check their sensitivity. In this section, we used SYBR-qPCR to analyze the different templates (including mycelial and sclerotial gDNA and standard DNA) from *R*. *solani*. Figure 3 shows the SYBR-qPCR results of the sensitivity tests using SMS RS-1F/SMS RS1-R and ITS1/GMRS-3. The PCR sensitivity levels of using SMS RS-1F/SMS RS1-R (Figures 3A,C,E) and ITS1/GMRS-3 (Figures 3B,D,F) to detect mycelial gDNA, standard DNA, and sclerotial gDNA were 10^–5^ ng, 10^2^ copies, and 10^–4^ ng, respectively. These results indicate that the sensitivity levels of the primers we designed, SMS RS-1F/SMS RS1-R, and that of the reference primer, ITS1/GMRS-3, are similar.

### Comparing the Molecular Detection Results From the Different Extraction Systems

Twenty-four samples with a score of 1 were collected from artificially Rs-inoculated samples. The DNA of their leaf sheaths was extracted using four DNA extraction methods (rapid DNA extraction, automatic magnetic bead-based DNA extraction, spin column-based DNA extraction, and CTAB/phenol/chloroform-based DNA extraction) and then analyzed by cPCR, SYBR-qPCR, and TaqMan-qPCR (Table 3). The detectable rates of ShB using rapid DNA extraction were 100 and 92% when using cPCR with the primers SMS RS1-F/SMS RS1-R and ITS1/GMRS-3, respectively, while they were 100 and 83%, respectively, when utilizing SYBR-qPCR with the same primers. However, the detectable rates of ShB using spin column-based DNA extraction were 100 and 88% when using cPCR with the primers SMS RS1-F/SMS RS1-R and ITS1/GMRS-3, respectively, while they were 96 and 75%, respectively, when utilizing SYBR-qPCR with the same primers. Furthermore, the detectable rates for all of them were 100% and the patterns were the same for automatic magnetic bead-based DNA extraction and CTAB/phenol/chloroform-based DNA extraction. Also, the rate was 100% when using TaqMan-qPCR with the primer set SMS RS1-F/SMS RS1-R.

**TABLE 3:** The molecular detection assays for artificially *Rhizoctonia solani*-inoculated samples using the molecular detection methods developed in this study.

**DNA extraction methods**	**Conventional PCR**		**TaqMan probe-based real-time PCR**	**SYBR green-based real-time PCR**	
	**SMS RS1-F/SMS RS1-R^e^**	**ITS1/GMRS-3^f^**	**SMS RS1-F/SMS RS1-R**	**SMS RS1-F/SMS RS1-R**	**ITS1/GMRS-3**
Rapid extraction^a^	24/24 (100%)	22/24 (92%)	24/24 (100%)	24/24 (100%)	20/24 (83%)
Automatic magnetic bead extraction^b^	24/24 (100%)	24/24 (100%)	24/24 (100%)	24/24 (100%)	24/24 (100%)
Column-based extraction^c^	24/24 (100%)	21/24 (88%)	24/24 (100%)	23/24 (96%)	18/24 (75%)
CTAB/phenol/chloroform-based extraction^d^	24/24 (100%)	24/24 (100%)	24/24 (100%)	24/24 (100%)	24/24 (100%)

*^*a*^The DNA extraction method was performed by using a rapid DNA extraction protocol developed in this study. ^*b*^The DNA extraction was performed by using a automatic silica-coated magnetic bead-based DNA extraction method (taco mini Automatic Nucleic Acid Extraction System, GeneReach, United States). ^*c*^The DNA extraction was performed by using a spin column-based DNA extraction method (Viogene genomic mini kit, Viogene-BioTek, Taipei, Taiwan). ^*d*^The DNA extraction was performed by a phenol/chloroform-based DNA extraction protocol according to Porebski et al. (1997). ^*e*^The *Rhizoctonia solani*-specific primer set SMS RS1-F/SMS RS1-R designed in this study was used for the molecular detection assays of rice sheath blight. ^*f*^The *Rhizoctonia solani*-specific primer set ITS1/GMRS-3 designed by Johanson et al. (1998) was used for comparison with the primer set SMS RS1-F/SMS RS1-R.*

### Comparing the Reproducibility of the Molecular Detection Systems

To be certain of the reproducibility of the molecular detection systems, DNA from samples with a score of 3 were extracted at four different times (intraday) and on four different days at the same time (interday) for three replicates. The cPCR, SYBR-qPCR, and TaqMan-qPCR were then used for analysis to calculate the coefficients of variation (CV) (Table 4). We chose three relatively convenient DNA extraction methods for these comparisons. As shown in Table 4, overall, all the detection rates of the cPCR for the diagnosis of ShB in the mildly symptomatic rice were good and similar. When using SYBR-qPCR for detecting the samples, the variability of the rates in detecting ShB when using the primer set SMS RS1-F/SMS RS1-R were better than the results obtained from using the primer set ITS1/GMRS-3 (Table 4). Moreover, the detection rates for the mildly symptomatic samples when using TaqMan-qPCR were better than the results obtained from using SYBR-qPCR (with primer set SMS RS1-F/SMS RS1-R) (Table 4). It is worth noting that the rates of variability in detecting ShB were relatively acceptable when using the rapid DNA extraction and automatic magnetic bead-based DNA extraction (Table 4).

**TABLE 4:** Intraday and interday validations of the molecular detections methods developed in this study.

**DNA extraction methods**	**Reproducibility assays (Coefficients of variation (CV), %)^d^**					
		**Conventional PCR**		**TaqMan probe-based real-time PCR**	**SYBR green-based real-time PCR**	
		**SMS RS1-F/SMS RS1-R^f^**	**ITS1/GMRS-3^g^**	**SMS RS1-F/SMS RS1-R**	**SMS RS1-F/SMS RS1-R**	**ITS1/GMRS-3**
Rapid DNA extraction^a^	Interday assays^e^	0.8 ± 1.0	0.4 ± 0.2	9.4 ± 5.6	18.4 ± 9.2	57.6 ± 37.9
	Intraday assays^e^	0.5 ± 0.4	0.6 ± 0.3	3.7 ± 2.1	10.6 ± 10.6	85.4 ± 10.0
Automatic magnetic bead-based DNA extraction^b^	Interday assays	0.2 ± 0.1	0.1 ± 0.2	9.6 ± 9.9	7.9 ± 8.8	9.3 ± 9.3
	Intraday assays	0.3 ± 01	0.3 ± 0.1	4.6 ± 1.4	6.6 ± 4.5	10.2 ± 5.3
Spin column-based DNA extraction^c^	Interday assays	1.1 ± 0.6	1.3 ± 0.4	5.4 ± 1.2	12.3 ± 11.1	23.9 ± 12.6
	Intraday assays	0.3 ± 0.2	0.5 ± 0.2	19.8 ± 21.9	40.9 ± 27.2	55.7 ± 39.0

*^*a*^The DNA extraction method was performed by using a rapid DNA extraction protocol developed in this study. ^*b*^The DNA extraction was performed by using a taco mini automatic silica-coated magnetic bead-based DNA extraction protocol (taco mini Automatic Nucleic Acid Extraction System, GeneReach, United States). ^*c*^The DNA extraction was performed by using a spin column-based DNA extraction protocol (Viogene genomic mini kit, Viogene-BioTek, Taipei, Taiwan). ^*d*^Coefficients of variation (CVs) are defined as standard deviations divided by the mean from each assay (*n* = 4) × 100%. ^*e*^Intra- (four assays on the same day) and interday- (four assays on different days) validations were carried out according to Skottrup et al. (2007) to evaluate experimental variation (CVs). ^*f*^The *Rhizoctonia solani*-specific primer set SMS RS1-F/SMS RS1-R designed in this study was used for the molecular detection assays of rice sheath blight. ^*g*^The *Rhizoctonia solani*-specific primer set ITS1/GMRS-3 designed by Johanson et al. (1998) was used for comparison with the primer set SMS RS1-F/SMS RS1-R.*

### Field Detection of ShB

The two relatively more reproducible DNA extraction methods, rapid DNA extraction and automatic magnetic bead-based DNA extraction, were used for ShB field detection, and their results were then compared with those for the traditional laboratory methods based on the use of plate-out assay for diagnosing ShB. A field detection evaluation was further performed to determine whether the molecular detection methods based on cPCR, SYBR-qPCR, and TaqMan-qPCR were suitable for the diagnosis of ShB. For this purpose, we collected a total of 96 rice leaf sheath samples (with symptom scale scores of 0 to 9) infected with Rs from 8 different fields that had been strongly affected by ShB for the plate-out assays and molecular detection assays. Utilizing PCR with rice heat shock protein primer 169A-Fw/169A-Rv (Guan et al., 2004) as an internal control, it was found that the success rates of the PCRs were all 100% regardless of the disease score (data not shown). As shown in Tables 5, 6, the detection rate of the plate-out assays for the diagnosis of ShB in the asymptomatic (score 0) to severely symptomatic (score 9) samples ranged from 0 to 94%, indicating that those molecular detection results for ShB were in agreement with the symptomatic characteristics and plate-out assay results. The detection rate for all the samples when using the primer set SMS RS1-F/SMS RS1-R was comparable to or better than that when using the published primer set ITS1/GMRS-3 (Tables 5, 6). As shown in Table 5, when using the rapid DNA extraction-based detection methods, the detection rates of cPCR, SYBR-qPCR, and TaqMan-qPCR with the primers SMS RS1-F/SMS RS1-R for the diagnosis of ShB in the asymptomatic (score 0)/mildly symptomatic (score 1) samples were 69%/100%, 100%/100%, and 100%/100%, respectively. As shown in Table 6, when using the automatic magnetic bead-based DNA extraction, the detection rates of cPCR, SYBR-qPCR, and TaqMan-qPCR with the primers SMS RS1-F/SMS RS1-R for the diagnosis of ShB in the asymptomatic (score 0)/mildly symptomatic (score 1) samples were 38%/88%, 100%/100%, and 100%/100%, respectively. These data supported the conclusion that using SYBR-qPCR and TaqMan-qPCR with the primers SMS RS1-F/SMS RS1-R is suitable for the detection of ShB in field-infected rice samples even if the infected rice samples only exhibit asymptomatic or mild symptoms. In addition, all the samples of infected rice yielded positive Rs detection results when using TaqMan-qPCR and SYBR-qPCR (SMS RS1-F/SMS RS1-R) with the automatic magnetic bead-based DNA extraction, indicating that the two molecular detection protocols were suitable for the field-diagnosis of ShB in all asymptomatic and symptomatic rice samples.

**TABLE 5:** The molecular detection of various *Rhizoctonia solani*-infected symptomatic samples using the rapid DNA extraction-based detection methods developed in this study.

**Symptoms of leaf sheath samples^a^**	**Plate-out assay^b^**	***In*-*planta* detection^b^**				
		**Conventional PCR**		**TaqMan probe-based real-time PCR**	**SYBR green-based real-time PCR**	
		**SMS RS1-F/SMS RS1-R^c^**	**ITS1/GMRS-3^d^**	**SMS RS1-F/SMS RS1-R**	**SMS RS1-F/SMS RS1-R**	**ITS1/GMRS-3**
Scale 0	0/16 (0%)	11/16 (69%)	8/16 (50%)	16/16 (100%)	16/16 (100%)	0/16 (0%)
Scale 1	8/16 (50%)	16/16 (100%)	16/16 (100%)	16/16 (100%)	16/16 (100%)	12/16 (75%)
Scale 3	11/16 (69%)	16/16 (100%)	16/16 (100%)	13/16 (81%)	15/16 (94%)	5/16 (31%)
Scale 5	13/16 (81%)	16/16 (100%)	11/16 (69%)	14/16 (88%)	16/16 (100%)	6/16 (38%)
Scale 7	15/16 (94%)	16/16 (100%)	15/16 (94%)	7/16 (44%)	12/16 (75%)	5/16 (31%)
Scale 9	15/16 (94%)	16/16 (100%)	13/16 (81%)	8/16 (50%)	10/16 (63%)	5/16 (31%)

*^*a*^The severity of sheath blight was scored with a scale of 0–9 based on relative lesion height on the whole plant according to IRRI (1996) and Rodrigues et al. (2003a, b) with minor modification, as follows: 0 = no symptom; 1 = lesions limited to the lower 20% of plant height; 3 = lesions limited to the lower 20–30% of plant height; 5 = lesions limited to the lower 31–45% of plant height; 7 = lesions limited to the lower 46–65% of plant height; and 9 = lesions on the upper 35% of plant height. ^*b*^A total of 96 varying symptomatic rice leaf sheath were used for plate-out assay and *in*-*planta* detection. ^*c*^The *Rhizoctonia solani*-specific primer set SMS RS1-F/SMS RS1-R designed in this study was used for the molecular detection assays of rice sheath blight. ^*d*^The *Rhizoctonia solani*-specific primer set ITS1/GMRS-3 designed by Johanson et al. (1998) was used for comparison with the primer set SMS RS1-F/SMS RS1-R.*

**TABLE 6:** The molecular detection of various *Rhizoctonia solani*-infected symptomatic samples using the automatic magnetic bead-based DNA extraction methods.

**Symptoms of leaf sheath samples^a^**	**Plate-out assay^b^**	***In*-*planta* detection^b^**				
		**Conventional PCR**		**TaqMan probe-based real-time PCR**	**SYBR green-based real-time PCR**	
		**SMS RS1-F/SMS RS1-R^c^**	**ITS1/GMRS-3^d^**	**SMS RS1-F/SMS RS1-R**	**SMS RS1-F/SMS RS1-R**	**ITS1/GMRS-3**
Scale 0	0/16 (0%)	6/16 (38%)	6/16 (38%)	16/16 (100%)	16/16 (100%)	10/16 (63%)
Scale 1	7/16 (44%)	14/16 (88%)	14/16 (88%)	16/16 (100%)	16/16 (100%)	16/16 (100%)
Scale 3	10/16 (63%)	16/16 (100%)	16/16 (100%)	16/16 (100%)	16/16 (100%)	16/16 (100%)
Scale 5	13/16 (81%)	15/16 (94%)	15/16 (94%)	16/16 (100%)	16/16 (100%)	15/16 (94%)
Scale 7	15/16 (94%)	16/16 (100%)	16/16 (100%)	16/16 (100%)	16/16 (100%)	16/16 (100%)
Scale 9	15/16 (94%)	16/16 (100%)	15/16 (94%)	16/16 (100%)	16/16 (100%)	16/16 (100%)

*^*a*^The severity of sheath blight was scored with a scale of 0–9 based on relative lesion height on the whole plant according to IRRI (1996) and Rodrigues et al. (2003a, b) with minor modification, as follows: 0 = no symptom; 1 = lesions limited to the lower 20% of plant height; 3 = lesions limited to the lower 20–30% of plant height; 5 = lesions limited to the lower 31–45% of plant height; 7 = lesions limited to the lower 46–65% of plant height; and 9 = lesions on the upper 35% of plant height. ^*b*^A total of 96 varying symptomatic rice leaf sheath were used for plate-out and *in*-*planta* detection assay. ^*c*^The *Rhizoctonia solani*-specific primer set SMS RS1-F/SMS RS1-R designed in this study was used for the molecular detection assays of rice sheath blight. ^*d*^The *Rhizoctonia solani*-specific primer set ITS1/GMRS-3 designed by Johanson et al. (1998) was used for comparison with the primer set SMS RS1-F/SMS RS1-R.*

## Discussion

In this study, we used ITS1/GMRS-3 as a reference (Johanson et al., 1998) to develop a specific primer set SMS RS1-F/SMS RS1-R for ShB detection. After testing them on ten samples of Rs (ShB), five samples of *P*. *oryzae* (rice blast), four samples of *B*. *oryzae* (rice brown spot), and nine samples with different fungal pathogens (Table 1), as well as on 96 field rice samples of ShB (Tables 5, 6), we can be sure that both primer sets have specificity for ShB. In addition, different molecular detection techniques, such as cPCR, SYBR-qPCR, and TaqMan-qPCR, were used to determine the sensitivity of both primer sets. Furthermore, both primer sets were used to test their sensitivity to mycelial genomic DNA, standard DNA, and sclerotial DNA of Rs, and achieved almost the same results in SYBR-qPCR (Figure 3). However, it was found that the primer set ITS1/GMRS-3 is not suitable for TaqMan-qPCR, as all the samples of rice infected with Rs yielded negative ShB detection results when TaqMan-qPCR was performed with the ITS1/GMRS-3 set (data not shown). All in all, the specificity and sensitivity results indicated that there are no differences between the two sets of primers since we used the ITS1/GMRS-3 set as a reference to design the SMS RS1-F/SMS RS1-R set.

In this study, we developed a rapid DNA extraction protocol for the on-site detection of ShB. Traditional extraction methods take lots of time, are costly, are not practically used (Fraczek et al., 2019); however, the method developed in this study addresses these shortcomings. The cost and time needed for each type of extraction approach are as follows: automatic magnetic bead-based DNA extraction, c.a. 3.5 USD/reaction (Yoon et al., 2016), 35 min (Ibrahim et al., 2018); spin column-based DNA extraction, c.a. 1.6–6.6 USD/reaction (Gupta and Preet, 2012), 30–120 min (Gupta and Preet, 2012); CTAB/phenol/chloroform-based extraction (organic solvent reaction method), c.a. less than 0.6 USD/reaction (Chen et al., 2010), 240 min (Gupta and Preet, 2012); the rapid extraction (single-reagent method) developed in this study, less than 0.03 USD, 3–5 min. Therefore, our single-reagent method requires less time and money than all the others, making it the most effective one in those terms.

To evaluate the effectiveness of this new method in extracting DNA from a plant with a pathogen score of 1, we tested all the extraction methods listed above and compared the results (Table 3). By using the single-reagent method to extract DNA, 100% positive reactions were observed when using the developed primer set, SMS RS1-F/SMS RS1-R, along with cPCR, SYBR-qPCR, or TaqMan-qPCR; however, only 92 and 83% positive reactions, respectively, were observed when using the reference primer set, ITS1/GMRS-3, along with cPCR and SYBR-qPCR. When using automatic magnetic bead-based extraction, all the tests achieved 100% positive reactions. When using a column extraction system, 100% positive reactions were achieved with the SMS RS1-F/SMS RS1-R set and cPCR or TaqMan-qPCR; however, 100% positive reactions were also achieved with the ITS1/GMRS-3 set and SYBR-qPCR. As for the organic solvent reaction method, as expected, all the samples yielded positive ShB detection results when using all the molecular detection techniques mentioned above. Although the single-reagent method may not achieve 100% positive reactions all the time, it is undoubtedly a convenient and economical way to detect the disease in the field. All the extraction methods mentioned herein can be used to identify the primary sickness of ShB, and can be used to determine whether a given plant has the disease pathogen or not.

The rice sheaths of infected plants with disease scores of 3 were subjected to interday and intraday assays to calculate the developed method’s variability and ensure its reproducibility. More specifically, lower levels of variability indicate better reproducibility (Table 4; Skottrup et al., 2007; Dixit et al., 2010). When detecting with cPCR plus the SMS RS1-F/SMS RS1-R and ITS1/GMRS-3 primers, the variations in results were between 0.2–1.1% and 0.1–1.3%, respectively (Table 4). It can thus be said that there is high reproducibility when using cPCR with these three extraction systems. However, when using SYBR-qPCR with SMS RS1-F/SMS RS1-R and ITS1/GMRS-3, the results were not as good, at 6.6–40.9% and 9.3–85.4%, respectively (Table 4). Furthermore, when using TaqMan-qPCR with SMS RS1-F/SMS RS1-R, the variations in the testing results ranged from 3.7 to 19.8% (Table 4). Overall, then, cPCR can be seen as the most stable way to detect moderately symptomatic rice (symptom score 3) since it has high reproducibility.

As for the extraction methods, with both SYBR-qPCR and TaqMan-qPCR, the automatic magnetic bead-based DNA extraction was the most stable method for extracting the DNA of Rs from rice (Table 6). In contrast, the rapid DNA extraction-based detection method may not be sufficiently stable since it cannot effectively remove other substances, e.g., polysaccharide (Krishnan and Hegde, 2018; Wang et al., 2019), protein (Wang et al., 2019), and phenol (Krishnan and Hegde, 2018; Wang et al., 2019). Since there are full of polysaccharides in plant’s cell, most of the regents may add CTAB to purify the DNA easily (Zhang and Stewart, 2000; Khan et al., 2004; Puchooa, 2004). From the results we tested, although there is no surfactant in extraction buffer, it can also extract DNA from rice tissue and receive a positive reaction in the molecular detection assays. However, since the absence of surfactants, the concentration of DNA is not as high as others (data not shown).

## Conclusion

Rice ShB, which is caused by *R*. *solani*, causes massive limitations on rice production nowadays (Kumar et al., 2019). In this study, we developed a fast and unique system to detect this disease in plants with different disease grades. The extracted DNA can be used with different testing methods, and it achieved good results with single buffer extraction, automatic magnetic bead-based DNA extraction, and column-based purification, as well as with interday and intraday assays. The *in-planta* testing results in this study also indicated that the developed extraction method can effectively detect sick plants, including even those that are not symptomatic. It is hoped, therefore, that the developed method can be used as a warning system for rice ShB in the near future.

## Data Availability Statement

The original contributions presented in the study are included in the article/supplementary material, further inquiries can be directed to the corresponding author.

## Author Contributions

Y-HL analyzed data, wrote the manuscript, and conceived, designed and supervised the project. S-MS, C-JW, and Y-JL performed the experiments. C-JW and T-DC revised the manuscript. S-CC provided some good suggestions for the subject. All authors discussed the data and read the manuscript.

## Conflict of Interest

The authors declare that the research was conducted in the absence of any commercial or financial relationships that could be construed as a potential conflict of interest.
